# Influence of Boiling, Steaming and Frying of Selected Leafy Vegetables on the In Vitro Anti-inflammation Associated Biological Activities

**DOI:** 10.3390/plants7010022

**Published:** 2018-03-16

**Authors:** K. D. P. P. Gunathilake, K. K. D. S. Ranaweera, H. P. V. Rupasinghe

**Affiliations:** 1 Department of Food Science & Technology, Faculty of Livestock, Fisheries & Nutrition, Wayamba University of Sri Lanka, Makandura, Gonawila 10250, Sri Lanka; gunathilakep@Dal.Ca; 2 Department of Food Science and Technology, Faculty of Applied Sciences, University of Sri Jayewardenepura, Gangodawila, Nugegoda 60170, Sri Lanka; kkdsran@yahoo.com; 3 Department of Plant, Food, and Environmental Sciences, Faculty of Agriculture, Dalhousie University, Truro, NS B2N 5E3, Canada

**Keywords:** plant-food, processing, nutraceuticals, inflammation, health

## Abstract

The aim of the present study was to evaluate the effect of cooking (boiling, steaming, and frying) on anti-inflammation associated properties *in vitro* of six popularly consumed green leafy vegetables in Sri Lanka, namely: *Centella asiatica*, *Cassia auriculata*, *Gymnema lactiferum*, *Olax zeylanica*, *Sesbania grnadiflora,* and *Passiflora edulis.* The anti-inflammation associated properties of methanolic extracts of cooked leaves were evaluated using four *in vitro* biological assays, namely, hemolysis inhibition, proteinase inhibition, protein denaturation inhibition, and lipoxygenase inhibition. Results revealed that the frying of all the tested leafy vegetables had reduced the inhibition abilities of protein denaturation, hemolysis, proteinase, and lipoxygenase activities when compared with other food preparation methods. Steaming significantly increased the protein denaturation and hemolysis inhibition in *O. zeylanica* and *P. edulis.* Steaming of leaves increased inhibition activity of protein denaturation in *G. lactiferum* (by 44.8%) and *P. edulis* (by 44%); hemolysis in *C. asiatica*, *C. auriculata,* and *S. grandiflora*; lipoxygenase inhibition ability in *P. edulis* (by 50%), *C. asiatica* (by 400%), and *C. auriculata* leaves (by 250%); proteinase inhibition in *C. auriculata* (100%) when compared with that of raw leaves. In general, steaming and boiling in contrast to frying protect the health-promoting properties of the leafy vegetables.

## 1. Introduction

Many degenerative human diseases, such as cancer, inflammation, and cardiovascular diseases have been recognized as a consequence of free radical damage. [1] Inflammation is a part of the complex biological response of vascular tissues to harmful stimuli, which is frequently linked with pain and involves many biological occurrences, such as an increase of vascular permeability, an increase of protein denaturation, and membrane alteration [2]. Numerous recent studies have shown that chronic inflammation is associated with a wide range of progressive diseases such as cancer, neurological disease, metabolic disorder, and cardiovascular disease [3]. Therefore, there have been many studies undertaken on how to delay or prevent the onset of these chronic diseases, as these lead to global health problems. The most likely and practical way to fight against degenerative diseases, such as inflammation, is to improve body antioxidant status, which could be achieved by higher consumption of vegetables and fruits [4]. Foods from plant origin usually contain natural antioxidants that can scavenge free radicals [5]. Green leafy vegetables are rich sources of minerals and antioxidant vitamins [6], as well as other antioxidant compounds, such as polyphenols and carotenoids [7,8] Among the leafy vegetables that are available in Sri Lanka, *Cassia auriculata*, *Gymnema lactiferum*, *Olax zeylanica*, *Sesbania grnadiflora*, *Passiflora edulis*, and *Centella asiatica* are reported to possess higher antioxidant activities, as described in Gunathilake and Ranaweera [7]. Although green leafy vegetables are considered as potential sources of dietary antioxidants and bioactives, only a few studies may have been reported on evaluating the impact of cooking on the anti-inflammatory properties. Therefore, this study aimed to investigate the influence of cooking of selected leafy vegetables on their anti-inflammation associated biological properties in vitro, which were measured by heat-induced hemolysis assay, protein denaturation assay, proteinase inhibition assay, and lipoxygenase inhibition assay.

## 2. Results and Discussion

### 2.1. Protein Denaturation

**Figure 1:**
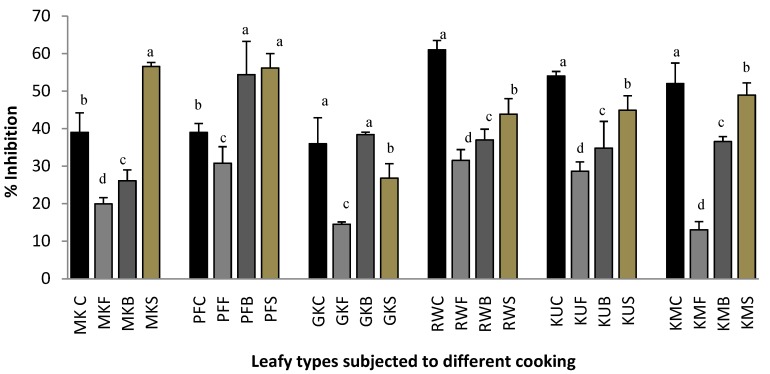
Protein denaturation inhibition ability of raw and cooked extracts of some GLV. MK, *O. zeylanica*; RW, *C. auriculata*; KM, *S. grandiflora*; KU, *G. lactiferum*; PF, *P. edulis*; GK, *C. asiatica*. C-fresh leaves; F-fried; B-boiled; S-steamed. Data are presented as means ± standard deviations of three replicate determinations. Columns with different letters for each vegetable are significantly different (*p* < 0.05).

Denaturation of proteins is a well-documented cause of inflammation [9]. As part of the investigation on the changes in the anti-inflammatory activity, the ability of cooked leaf extracts on protein denaturation was studied. Figure 1 shows the effect of cooking of leafy vegetables on protein denaturation inhibitory activity. Protein denaturation inhibition ability significantly increased (*p* < 0.05) in steamed leaves of *O. zeylanica* (by 44.8%) and *P. edulis* (by 44%) when compared with that of their fresh leaves. Interestingly, frying of all the tested leafy vegetables resulted in the lowest protein denaturation inhibition ability when compared with other processing methods. Boiled leaves of *O. zeylanica*, *C. auriculata*, *S. grandiflora,* and *G. lactiferum* have shown significantly lower (*p* < 0.05) inhibition ability when compared with their fresh leaves, whereas boiled leaves of *C. asiatica*, and *P. edulis* have shown a significantly higher (*p* < 0.05) protein denaturation inhibition ability. Significantly lower protein denaturation inhibition ability was observed in all of the cooked leaves of *C. auriculata*, *S. grandiflora,* and *G. lactiferum* when compared with their fresh leaves. In a study, methanol extract of whole plant of *Oxalis corniculata* Linn (Family: Oxalidaceae) was assessed by Sakat et al. [9] for its anti-inflammatory activity by in vitro methods and was reported that the extract showed anti-inflammatory activity by inhibiting the heat-induced albumin denaturation with the IC_50_ values of 288.04 ± 2.78 µg/mL, respectively. Based on another study, a flavonoid-rich fraction of *M. myristica* have shown an albumen denaturation in a dose-dependent manner and the maximum inhibition of denaturation of albumin was found to around 75.38% ± 0.56% at 350 μg/mL: IC_50_ value of 258 μg/mL, while a standard anti-inflammatory drug (aspirin) showed a maximum inhibition of 98.41% ± 0.13% at the same concentration [10]. However, the precise mechanism of this membrane stabilization was yet to be elucidated. It has been proposed that the plant extracts might inhibit the release of the lysosomal content of neutrophils at the site of inflammation [11]. According to Chou [12], these neutrophils lysosomal constituents include bactericidal enzymes and proteinases which upon extracellular release cause further tissue inflammation and damage. 

Table 1 shows the correlation of anti-inflammatory properties with polyphenols, flavonoids, and carotenoids of green leafy vegetables. The changes in protein denaturation ability with the cooking treatments may be related to the changes in polyphenols and flavonoids content. In a study, it was found that heating of green leafy vegetables reduced the vitamin C content, thus reducing properties and free radicals scavenging properties [5].

### 2.2. Heat-Induced Hemolysis

According to Chippada and co-authors [13], lysosomal enzymes that are released during inflammation leads to the tissue injury by damaging the macromolecules, such as proteins, lipids, DNA, etc. Further, it damages tissues by lipid peroxidation of membranes, which are assumed to be responsible for certain pathological conditions as heart attacks and rheumatoid arthritis, etc. Therefore, the stabilization of lysosomal membrane is vital in controlling the inflammatory response by inhibiting the release of lysosomal constituents of activated neutrophil, such as bactericidal enzymes and proteases, which may lead to further tissue inflammation and damage upon extracellular release or by stabilizing the lysosomal membrane [13]. The membrane of the human’s red blood cell is analogous to the lysosomal membrane, and its stabilization implies that the extract may as well stabilize lysosomal membranes. The in vitro bioassay that was used in this study determines the stabilization of human red blood cell membrane by hypo tonically induced membrane lysis, and this can be taken as an in vitro measure of anti-inflammatory activity of the many drugs or various plant extracts [13]. 

**Figure 2:**
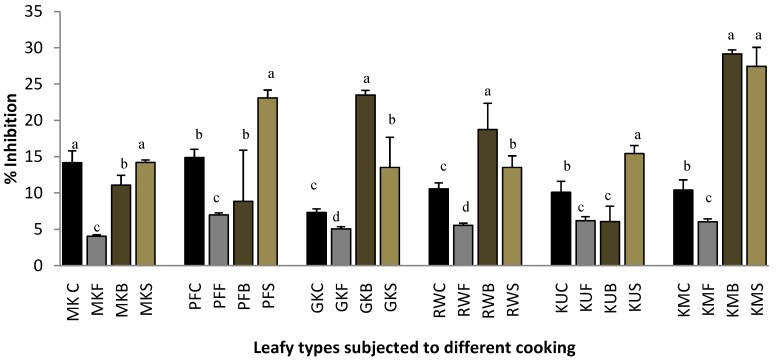
Heat-induced hemolysis inhibition ability of raw and cooked extracts of some GLV. Values represent means of triplicate readings. MK, *O. zeylanica*; RW, *C. auriculata*; KM, *S. grandiflora*; KU, *G. lactiferum*; PF, *P. edulis*; GK, *C. asiatica*. C-fresh leaves; F-fried; B-boiled; S-steamed. Data are presented as means ± standard deviations of three replicate determinations. Columns with different letters for each vegetable are significantly different (*p* < 0.05).

The heat-induced hemolysis inhibition abilities of raw and cooked leaf samples are shown in Figure 2. Similarly, the frying process significantly reduced (*p* < 0.05) the hemolysis inhibition ability of *O. zeylanica*, *C. auriculata*, *S. grandiflora*, *G. lactiferum*, *P edulis,* and *C. asiatica* by 4.0%, 5.5%, 6.0%, 6.2%, 7.0%, and 5.1%, respectively. Boiled leaves of *C. asiatica*, *C. auriculata,* and *S. grandiflora* have shown a significantly higher (*p* < 0.05) hemolysis inhibition ability hen compared with that of raw and their other cooked leaves. Steamed leaves of *O. zeylanica*, *P. edulis,* and *G. lactiferum* have shown the similar or higher hemolytic inhibition ability than that of their raw and other cooked leaves. Steamed and boiled leaves of *S. grandiflora* showed significantly higher (*p* < 0.05) hemolytic inhibition ability when compared with that of its raw leaves. In a previous study, the methanolic extract of the whole plant of *Oxalis corniculata* Linn has been shown the red blood cells membrane stabilization with the IC_50_ values of 467.1 ± 9.6 µg/mL [9]. Table 1 shows a poor correlation of heat-induced hemolysis with polyphenols, flavonoids, and carotenoids of green leafy vegetables.

### 2.3. Lipoxygenase Inhibitory Activity.

**Figure 3:**
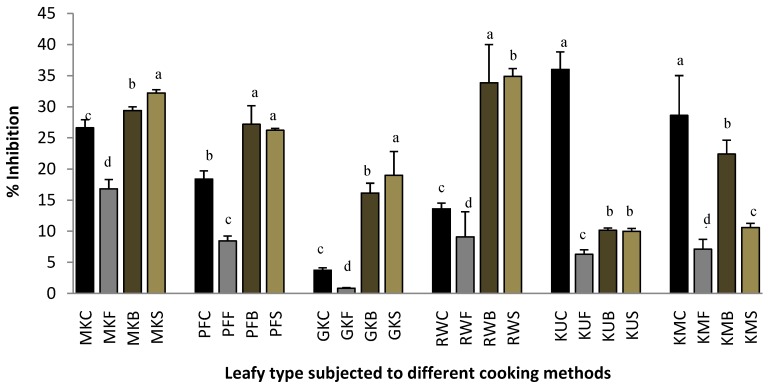
Lipoxygenase inhibition ability of raw and cooked extracts of some GLV. Values represent means of triplicate readings. MK, *O. zeylanica*; RW, *C. auriculata*; KM, *S. grandiflora*; KU, *G. lactiferum*; PF, *P. edulis*; GK, *C. asiatica*. C-fresh leaves; F-fried; B-boiled; S-steamed. Data are presented as means ± standard deviations of three replicate determinations. Columns with different letters for each vegetable are significantly different (*p* < 0.05).

The mechanism of anti-inflammation involves a series of events in which metabolism of Arachidonic acid plays an important role [10] Arachidonic acid is cleaved from the membrane phospholipids upon appropriate stimulation of neutrophils, and can be converted to leukotrienes and prostaglandins through the action of lipoxygenase and cyclooxygenase pathways, respectively [10]. Lipoxygenase enzymes catalyze the oxidation of Arachidonic acid (linoleic acid) to produce leukotrienes that are important mediators in a variety of inflammatory events [14]. In a previous study, it was reported that the essential oil of *Cymbopogon giganteus* from Benin has potential to be used as an anti-inflammatory agent towards lipoxygenase inhibition [14]. Therefore, use of in vitro inhibition of lipoxygenase could be a good model for the screening of plants with inflammatory potentials. Figure 3 shows the lipoxygenase inhibition ability of raw and cooked leafy types, and the results clearly showed that the lipoxygenase inhibition ability had reduced during frying in all of the leaf varieties when compared with their fresh, boiled, and steamed leaves. Boiling of leaves increased the lipoxygenase inhibition ability significantly (*p* < 0.05) in *O. zeylanica*, *P. edulis*, *C. asiatica,* and *C. auriculata* leaves when compared with that of their raw leaves. However, boiling has increased the lipoxygenase inhibition ability in *S. grandiflora* as compared with its steamed leaves, though it is lower than its fresh leave. Interestingly, cooked leaves of *G. lactiferum* have shown significantly lower (*p* < 0.05) lipoxygenase inhibition ability when compared with that of its raw leaves.

### 2.4. Proteinase Inhibitory Activity

Plant extracts have been reported to inhibit protein denaturation. Although, the precise mechanism of this membrane stabilization was yet to be elucidated, but it has been proposed that the extract might inhibit the release of the lysosomal content of neutrophils at the site of inflammation [9]. These neutrophils lysosomal constituents include bactericidal enzymes and proteinases, which, upon extracellular release, cause further tissue inflammation and damage [12]. 

**Figure 4:**
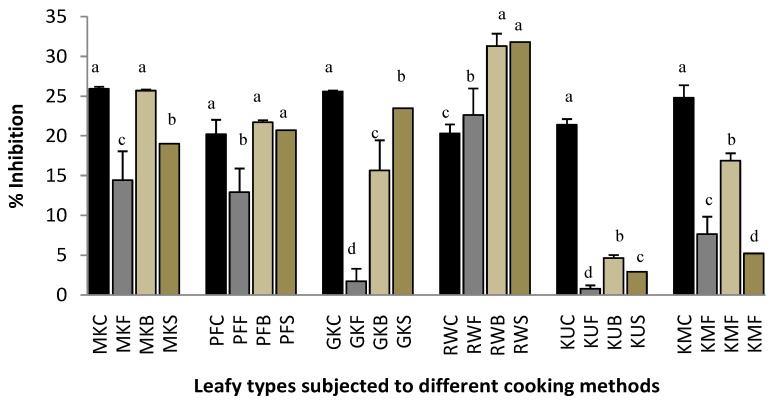
Proteinase inhibition ability of raw and cooked extracts of some GLV. Values represent means of triplicate readings. MK, *O. zeylanica*; RW, *C. auriculata*; KM, *S. grandiflora*; KU, *G. lactiferum*; PF, *P. edulis*; GK, *C. asiatica*. C-fresh leaves; F-fried; B-boiled; S-steamed. Data are presented as means ± standard deviations of three replicate determinations. Columns with different letters for each vegetable are significantly different (*p* < 0.05).

Figure 4 demonstrates the proteinase inhibition ability of raw and cooked leafy types. Fried leaves of *P. edulis*. *C. asiatica,* and *G. lactiferum* showed significantly lower (*p* < 0.05) inhibition % when compared with that of their raw, boiled, and steamed leaves. Raw leaves of *G. lactiferum* and *S. grandiflora* have shown higher proteinase inhibition ability compared with their all cooked forms. However, all of the cooked leaves of *C. auriculata* have shown similar or higher proteinase inhibition ability compared with that of its raw leaves. For examples, boiling and steaming of leaves of *C. auriculata*, increased the proteinase inhibitory activity by 54.2% and 56.7%, respectively. Boiled leaves of *O. zeylanica* exhibited a similar ability of proteinase inhibition to its raw leaves. However, fried and steamed leaves of *O. zeylanica* showed significantly lower (*p* < 0.05) proteinase inhibition ability when compared with that of boiled and raw leaves. About 80% reduction in proteinse inhibitory activity was observed in *Gymnema lactiferum*. According to the Table 1, anti-inflammatory associated activities are correlated (<0.5) with polyphenols, carotenoids, and flavonoids of green leafy vegetables. Therefore, the changes in proteinase inhibition ability with the cooking treatments may be related to the changes in polyphenols, flavonoids, and carotenoids content in these leaves.

In this study, it was found that boiling and steaming process might increase or decrease the anti-inflammatory activities. The frying process reduces the anti-inflammatory activity of all leafy types. The changes in anti-inflammatory activities of these studied leafy types in the cooking process may be due to changes in bioactives, such as polyphenols, flavonoids, and carotenoids. Wachtel-Galor and co-authors [15] have reported that an increase in polyphenols, such as flavonoids, after subsequent boiling or steaming, may be related to an enhanced availability for extraction, to a more efficient release of polyphenols or flavonoids compounds from intracellular proteins and altered cell wall structures. However, according to Palermo et al. [16], the more intense the cooking treatment, such as frying, the greater the flavonoid degradation. Further, High frying temperatures, in fact, could cause the oil to produce hydroperoxide free radicals and accelerate the degradation of carotenoids, as well lead to a reduction in their bioactivity [17]. Accordingly, variation in losses and gains of phenolics like bioactives due to cooking treatments in studied leafy types could be due to the types of cooking, the nature of leaves, and the forms of the bioactives that are present in the plant matrices.

## 3. Materials and Methods

### 3.1. Materials

Fresh green leafy vegetable samples; Ranawara (*Cassia auriculata)*, mella (*Olax Zeylanica)*, Gotukola (*Centella asiatica)*, Ceylon cow tree (*Gymnema lactiferum)*, Kathurumurunga (*Sesbania grandiflora),* and Passion fruit (*Passiflora edulis*) were collected in Gampaha and Kurunegala districts in Sri Lanka. All of the chemicals were of analytical grade and were purchased from Sigma Aldrich, St. Louis, MO, USA through Analytical Instrument Pvt Ltd., Colombo, Sri Lanka.

### 3.2. Preparation of Cooked Samples

Cleaned leaf samples were subjected to different cooking treatments separately at atmospheric pressure. Cooking conditions were selected based on preliminary trials. The cleaned and washed leaves were cut into small pieces, and the samples (400 g) were divided into four parts (100 g each), keeping one portion as control (uncooked, stored at 4 °C in the refrigerator until use for within 24 h), and the rest was subjected to different cooking treatments, as indicated below. Briefly, for boiling, leaf samples (100 g) were added to boiling tap water (150 mL) in a covered stainless-steel pot and were cooked on a moderate flame for 5 min, and then samples were drained off and cooled rapidly on plenty of ice. For steaming, leaf samples were placed on a perforated tray in a stainless steel steamer covered over boiling water for 5 min, and then samples were rapidly cooled on ice. For frying, leaves were added to 500 mL of white coconut oil (“N Joy”, Adamji and Lukmangi pvt Ltd., Colombo, Sri Lanka) in a stainless steel pan at 170 °C and stirred for three minutes until the sample became crisp-tender. At the end of each trial, the samples were drained off and dabbed with blotting paper to allow for the absorption of exceeding oil. The cooked leaf samples were homogenized and stored at −18 °C. As anti-inflammatory activities were calculated according to dry matter basis, moisture contents of the cooked samples were determined according to the method described by Turkmen et al. [18]. 

### 3.3. Preparation of Methanolic Extracts. 

Methanolic extracts of leaves were prepared according to the method described by Gunathilake & Ranaweera [8]. Briefly, one gram of cooked leafy vegetable samples was weighed and mixed with 8mL of 70% methanol and vortexed at high speed for thirty minutes, and then centrifuged (Hettich, EBA 20, Hettich GmbH & Co., Tuttlingen, Germany) for 10 min at 792 g. The extracts were subsequently filtered through a filter paper (Whatman No. 42, Whatman Paper Ltd, Maidstone, UK. The solvents that remained in crude extracts were removed using a rotary evaporator (HAHNVAPOR, Model HS-2005 V, HAHNSHIN Scientific, Seoul, Korea) at 40 °C. The prepared concentrated extracts were oven dried at 40 °C for 12 h and were stored at −18 °C in air-tight screw-capped cryogenic vials until they assayed within one week. Extracts were dissolved in methanol to obtain a concentration of 3 mg/mL for each assay. 

### 3.4. Anti-Inflammatory Properties

#### 3.4.1. Heat-Induced Hemolysis

Erythrocytes suspension was prepared by the method described by Shinde et al. [19], with some modifications [20]. Briefly, blood was obtained from a healthy human volunteer and transferred to heparinized centrifuge tubes and centrifuged at 3000 rpm for 5 min and washed three times with equal volume of normal saline (0.9% sodium chloride). The volume of the blood was measured and reconstituted as a 10% (*v*/*v*) suspension with isotonic buffer solution (10 mM sodium phosphate buffer pH 7.4, the composition of the buffer solution (g/L) was NaH_2_PO_4_ (0.2), Na_2_HPO_4_ (1.15) and NaCl (9.0). Heat-induced hemolysis was carried out, as described by Okoli et al. [21], with some modifications. About 0.05 mL of blood cell suspension and 0.05 mL extracts of cooked leaves were mixed with 2.95 mL phosphate buffer (pH 7.4), and the mixture was mixed gently and incubated at 54 °C for 20 min in a water bath. At the end of the incubation, the reaction mixture was centrifuged at 2500 rpm for 3 min and the absorbance of the supernatant measured at 540 nm using a UV/VIS spectrometer (Optima, SP-3000, Tokyo, Japan). Phosphate buffer solution without sample was used as the control. The level of hemolysis was calculated using the following relation Equation (1):

% inhibition of hemolysis = 100 × (1 − A2/A1)(1)

where, A1 = Absorption of the control sample, A2 = Absorption of test sample solution.

#### 3.4.2. Effect on Protein Denaturation 

The test was performed following the method described by Gambhire et al. [22], with some modifications [20]. Briefly, 0.2 mL of 1% bovine albumin, 4.780 mL of phosphate buffered saline (PBS, pH 6.4), and 0.02 mL of cooked leaf extract was mixed gently, and was incubated at 37 °C for 15 min in a water bath, and then the reaction mixture was heated at 70 °C for 5 min. After cooling, the absorbance of the solutions was measured at 660 nm using a UV/VIS spectrometer. Phosphate buffer solution without sample was used as the control, and the percentage inhibition of protein denaturation was calculated by using the following formula Equation (2)

% inhibition of denaturation = 100 × (1 − A2/A1)(2)

where A1 = Absorption of the control sample and A2 = Absorption of the test sample 

#### 3.4.3. Proteinase Inhibitory Activity

The test was performed according to the modified method of Sakat et al. [9], with some modifications, as suggested by [20]. Briefly, 0.06 mg trypsin, 1 mL of 20 mm Tris-HCl buffer (pH 7.4), 0.02 mL cooked leaf extract, and 0.980 mL methanol were mixed, and the reaction mixture was incubated at 37 °C for 5 min, and then 1 mL of 0.8% (*w*/*v*) casein was added. The mixture was incubated further for an additional 20 min. About 2 mL of 70% perchloric acid was added to terminate the reaction. Cloudy suspension was centrifuged, and the absorbance of the supernatant was read at 210 nm against buffer as blank. The percentage of inhibition of proteinase activity was calculated.

Phosphate buffer solution without sample was used as the control. The percentage inhibition of protein denaturation was calculated by using the following Equation (3):

% inhibition of denaturation = 100 × (1 − A2/A1)(3)

where A1 = Absorption of the control sample and A2 = Absorption of the test sample

#### 3.4.4. Lipoxygenase Inhibition Assay

Lipoxygenase was assayed, according to the method described by Wu [23], with some modifications being mentioned in Gunathilake [20]. Briefly, 1 mL sodium borate buffer (0.1 M, pH 8.8) and 10 μL lipoxygenase (8000 U/mL) was incubated with 10 μL cooked leaf extract in a 1 mL cuvette at room temperature for 5 min. The reaction was started by the addition of linoleic acid substrate (10 μL, 10 mmol). The absorbance of the resulting mixture was measured at 234 nm, and the phosphate buffer solution without sample was used as the control, and the percentage inhibition of lipoxygenase was calculated using the following Equation (4):

% Inhibition = 100 × (absorbance of the control − absorbance of the sample)/absorbance of the control(4)

### 3.5. Analysis of Phenolics, Flavonoids and Carotenoids 

Analysis of polyphenols was measured, as described in Gunathilake and Rupasinghe [24], flavonoid content using Gunathilake et al. [6], and the carotenoid content by using the method that was described in Gunathilake and Ranaweera [8]. However, data are not shown in this paper, and the data were used for the correlation studies with anti-inflammatory data.

### 3.6. Statistical Analysis

All data are presented as the mean ± standard deviation for all in vitro assays done. All of the samples were analyzed in triplicate, and one-way analysis of variance (ANOVA) was performed using MINITAB 15 software (Minitab Inc, State College, PA, USA). When there were significant differences (*p* < 0.05), multiple mean comparisons were carried out using LSD method. Pearson’s correlation coefficients (*r*), with the level of significance (*P ≤* 0.05) (2-tailed) for total polyphenols, flavonoids, and carotenoids versus studied anti-inflammatory results were estimated using MINITAB 15 software. Polyphenols, flavonoids, and carotenoids content of the extracts that were used for correlation studies are based on the same study; however, data are not shown.

## 4. Conclusions

The present study clearly indicates that the in vitro anti-inflammatory associated biological activities of studied green leafy vegetables are modified, increased or decreased, by boiling, steaming, and frying process, depending upon the vegetable species. Among the cooking methods, the frying of all leafy vegetables has reduced the inhibition abilities of protein denaturation, hemolysis, proteinase, and lipoxygenase activities when compared with other cooking methods that were studied. Steaming significantly increased the protein denaturation and hemolysis inhibition in *O. zeylanica* and *P. edulis.* Boiling of leaves increased the inhibitory activity of protein denaturation in *C. asiatica* and *P. edulis*; hemolysis in *C. asiatica*, *C. auriculata,* and *S. grandiflora*; lipoxygenase inhibition ability in *O. zeylanica*, *P. edulis*, *C. asiatica* and *C. auriculata* leaves; proteinase inhibition in *C. auriculata* when compared with that of raw and their other cooked leaves. The results of the study can be used as a database, providing information on the effects of different cooking methods on the health promotion potential of green leafy vegetables studied.

## Figures and Tables

**Table 1:** Pearson correlations between major bioactives (total phenolics, total flavonoids, total carotenoids) and % inhibition of protein denaturation, hemolysis denaturation, lipoxygenase activity, and proteinase activity of cooked leafy vegetables.

Parameters	*r*	*P*
Total phenolics versus protein denaturation	0.646	0.001
Total phenolics versus hemolysis	0.294	0.024
Total phenolics versus lipoxygenase activity	0.558	0.000
Total phenolics versus proteinase activity	0.594	0.001
Total flavonoids versus protein denaturation	0.519	0.000
Total flavonoids versus hemolysis	0.444	0.000
Total flavonoids versus lipoxygenase activity	0.592	0.001
Total flavonoids versus proteinase activity	0.666	0.000
Total carotenoids versus protein denaturation	0.106	0.420
Total carotenoids versus hemolysis	0.203	0.120
Total carotenoids versus lipoxygenase activity	0.564	0.000
Total carotenoids versus proteinase activity	0.634	0.000
